# Supraspinal Shaping of Adaptive Transitions in the State of Functional Connectivity Between Segmentally Distributed Dorsal Horn Neuronal Populations in Response to Nociception and Antinociception

**DOI:** 10.3389/fnsys.2019.00047

**Published:** 2019-09-26

**Authors:** Mario Martín, Javier Béjar, Diógenes Chávez, Adrian Ramírez-Morales, Edson Hernández, Leonardo Moreno, Enrique Contreras-Hernández, Silvio Glusman, Ulises Cortés, Pablo Rudomin

**Affiliations:** ^1^BarcelonaTech, Universitat Politècnica de Catalunya, Catalonia, Spain; ^2^Barcelona Supercomputing Center, Catalonia, Spain; ^3^Department of Physiology, Biophysics and Neurosciences, Center for Research and Advanced Studies, National Polytechnic Institute, Mexico City, Mexico; ^4^Stroger Cook County Hospital, Chicago, IL, United States; ^5^El Colegio Nacional, Mexico City, Mexico

**Keywords:** cord dorsum potentials, dorsal horn neurons, functional connectivity, machine learning, Markov process, state transitions, nociception, antinociception

## Abstract

In the anesthetized cat the correlation between the ongoing cord dorsum potentials (CDPs) recorded from different lumbar spinal segments has a non-random structure, suggesting relatively stable patterns of functional connectivity between the dorsal horn neuronal ensembles involved in the generation of these potentials. During the nociception induced by the intradermic injection of capsaicin, the patterns of segmental correlation between the spontaneous CDPs acquire other non-random configurations that are temporarily reversed to their pre-capsaicin state by the systemic injection of lidocaine, a procedure known to decrease the manifestation of neuropathic pain in both animals and humans. We have now extended these studies and utilized machine learning for the automatic extraction and selection of particular classes of CDPs according to their shapes and amplitudes. By using a Markovian analysis, we disclosed the transitions between the different kinds of CDPs induced by capsaicin and lidocaine and constructed a global model based on the changes in the behavior of the CDPs generated along the whole set of lumbar segments. This allowed the identification of the different states of functional connectivity within the whole ensemble of dorsal horn neurones attained during nociception and their transitory reversal by systemic administration of lidocaine in preparations with the intact neuroaxis and after spinalization. The present observations provide additional information on the state of self-organized criticality that leads to the adaptive behavior of the dorsal horn neuronal networks during nociception and antinociception both shaped by supraspinal descending influences.

## Introduction

Previous work has shown that in the anesthetized cat the spontaneous cord dorsum potentials (CDPs) recorded in a given lumbar segment have different shapes and amplitudes and may appear synchronized with potentials generated in other spinal segments (Contreras-Hernández et al., 2018). The correlation matrix between the CDPs recorded from different segments had a non-random structure, suggesting relatively stable patterns of functional connectivity between the dorsal horn neuronal ensembles involved in the generation of these potentials. During the chemical nociception induced by the intradermic injection of capsaicin, the patterns of segmental correlation between the spontaneous CDPs were reorganized and acquired other non-random configurations that were temporarily reversed to their pre-capsaicin state by the systemic injection of a small dose of lidocaine, a procedure known to reduce neuropathic pain in humans.

At present, we have limited information on the identity of the neuronal populations underlying the changes in the correlation between the spontaneous CDPs induced by nociceptive stimulation and their reversal by lidocaine. While searching for intermediate nucleus interneurons mediating presynaptic inhibition (Rudomin et al., 1987), we found one set of neurones (Type I) whose activity was preceded by negative CDPs (nCDPs) and another set (Type II) that was instead preceded by negative-positive CDPs (npCDPs). The activation of type I neurones led to the generation of short-lasting glycinergic inhibitory potentials in motoneurones, while the activation of Type II neurones was instead associated with the generation of inhibitory GABAergic potentials in motoneurones and with primary afferent depolarization (PAD) and presynaptic inhibition.

We initially assumed that separate populations of dorsal horn neurones generated the nCDPs and npCDPs (Rudomin et al., 1987). However, subsequent work has indicated that depending on the magnitude of the ongoing neuronal synchronization, nCDPs and npCDPs could be generated by the same population of dorsal horn neurones (Contreras-Hernández et al., 2015). During low levels of synchronization, activation of the dorsal horn neuronal ensemble would mainly generate nCDPs, and there would be a concurrent activation of the pathways mediating non-reciprocal glycinergic post-synaptic inhibition. In contrast, during higher states of neuronal synchronization, the activation of the same set of dorsal horn neurones would lead to the generation of npCDPs and to a preferential activation of the pathways mediating a GABAa PAD and presynaptic inhibition.

These observations indicated that the intrinsic patterns of functional connectivity between the populations of dorsal horn neurones determines their interaction with other neuronal networks. Yet, several questions remained to be addressed, among them, (a) how the functional connectivity of the neuronal populations generating the different classes of spontaneous CDPs changes after nociception and antinociception, (b) how the concurrent changes in neuronal connectivity affect information transmission and organization in sensory and motor pathways, and (c) which neurones are involved in the generation of the different classes of spontaneous CDPs, in addition to the nCDPs and npCDPs.

To approach the first two questions we utilized Machine Learning procedures that use similarity criteria for the automatic selection and classification of the ongoing CDPs according to their shape and amplitude (see Béjar et al., 2015; Martin et al., 2015). We used this procedure to build dictionaries with CDPs selected under basal (control) conditions to estimate the changes produced during nociception and antinociception on the probabilities of occurrence of the different classes of CDPs, a task that would be otherwise difficult to achieve using predetermined template selection methods such as those employed in previous studies (see Chávez et al., 2012; Contreras-Hernández et al., 2015).

The present study shows that in preparations with intact neuroaxis, the intradermic injection of capsaicin reduced the fractional probabilities of occurrence of most of the classes comprising the small amplitude CDPs and increased the probabilities of the largest CDPs. Following the systemic injection of a small dose of lidocaine, the different classes of CDPs temporarily displayed, rather closely, their fractional probabilities of occurrence attained before the nociceptive stimulation.

The changes displayed by the different classes of CDPs during the action of capsaicin and lidocaine were largely attenuated in previously spinalized preparations, a finding suggesting that supraspinal influences shape the activation and adaptability of spinal networks in response to nociception.

By using a Markovian analysis, we further estimated the transitions between the different kinds of CDPs induced by capsaicin and lidocaine and constructed a global model based on the changes in the behavior of the CDPs generated along the whole set of lumbar segments. This allowed the identification of the different states of functional connectivity within the whole ensemble of dorsal horn neurones attained during nociception and after the systemic administration of lidocaine in preparations with the intact neuroaxis as well as in previously spinalized preparations. These observations provide additional evidence pertaining the role of supraspinal influences in the shaping of the functional connectivity between dorsal horn neurones, a process of significance for information transmission and processing in the spinal cord. A previous account of these findings has been presented in Rudomin et al. (2016).

## Materials and Methods

### General Procedures

The data analyzed in this report were obtained from experiments included in a previous study (Contreras-Hernández et al., 2018) performed in adult cats of both sexes weighting between 2.4 and 4.5 Kg, initially anaesthetized with pentobarbitone sodium (40 mg/kg i.p.). During the dissection additional doses of pentobabitone sodium (5 mg/kg/h) were given intravenously to maintain an adequate level of anesthesia, tested by assessing that withdrawal reflexes were absent, that the pupils were constricted and that systolic arterial blood pressure was between 100 and 120 mm Hg.

The lumbosacral and low thoracic spinal segments were exposed by laminectomy and opening of the dura mater. After the primary surgical procedures, the animals were transferred to a stereotaxic metal frame allowing immobilization of the head and spinal cord and pools were made with the skin flaps that were filled with paraffin oil to prevent desiccation of the exposed tissues. The temperature was maintained between 36 and 37°C using radiant heat.

Subsequently, the animals were paralyzed with pancuronium bromide (0.1 mg/kg) and artificially ventilated. The tidal volume was adjusted to maintain 4% of CO_2_ concentration in the expired air. During paralysis, adequacy of anesthesia was ensured with supplementary doses of anesthetic (2 mg/kg in an hour) and by repeatedly assessing that the pupils remained constricted and that heart rate and blood pressure were not changed following a noxious stimulus (paw pinch).

Cord dorsum potentials were simultaneously recorded by means of 8–12 silver ball electrodes placed on the surface of the *L*4–*L*7 segments on both sides of the spinal cord using separate preamplifiers (bandpass filters 0.3 Hz to 1 kHz), visualized on-line and digitally stored for further analysis with software written in MatLab (MathWorks) and LabView version 14 (National Instruments).

As described by Rudomin and Hernández (2008), 30 μl of 1% solution of capsaicin diluted in 10% Tween 80 and 90% saline (around 7.5 μg/kg) were injected in the plantar cushion of the left hindlimb. To avoid desensitization, capsaicin was injected only once (Sakurada et al., 1992). The effects of capsaicin started around 10–20 min after injection, attained maximum values between 100 and 180 min and persisted up to 4 h. The injection of capsaicin produced a clear inflammatory response around the injection site (see Rudomin and Hernández, 2008).

In this series of experiments, a solution of Lidocaine (5 mg/kg diluted in 6 cc of isotonic saline) was slowly injected (20–30 min) through a separate catheter inserted in the right femoral vein. We used systemic application of lidocaine because this is the procedure that has been successfully used both clinically and experimentally to reduce neuropathic pain and preemptive analgesia (see Woolf and Chong, 1993; Kissin, 2000).

The usual procedure was to make control recordings of the spontaneous CDPs during 30–60 min that were followed by recordings made after the intradermic injection of capsaicin into the footpad of the left hindlimb and also after the systemic administration of lidocaine. Spinalization was performed by bathing the exposed *T4* segment with chilled Ringer solution for about 10 min, spraying it with liquid nitrogen until it was completely frozen and sectioned thereafter. At the end of the experiment, the animal was euthanized with a pentobarbital overdose and perfused with 10 p.c. formalin. The spinal cord was removed for fixation and dehydration to examine the completeness of the spinal sections.

### Data Processing

#### Extraction of Spontaneous CDPs With Specific Shapes and Amplitudes

To extract the CDP sequences we used the Machine Learning method described in a previous study (Martin et al., 2015). To this end, the whole procedure was divided into several steps that included: (a) the extraction of the CDPs from the raw recordings, (b) the classification and discretization of the selected CDPs, and (c) the analysis of their behavior at different levels of granularity in the spatial and temporal domain. This included the construction of histograms to display the fractional probability of occurrence of each class of the selected CDPs relative to the total number of CDPs extracted from a fixed time window (5–10 min). See Appendix for further details.

#### Sequential Behavior of the CDPs Recorded in Lumbar Segments

We have previously shown (Martin et al., 2015) that the dictionaries made with the CDPs extracted from control recordings made in different experiments were relatively stable during prolonged time periods (30–60 min). We also found that in a given segment, each time set had a specific dynamical behavior that was changed by the intradermic injection of capsaicin as well as by spinalization.

In order to capture these differences and to obtain a high-level description of the changes in the CDPs occurring during the experiment in different spinal segments, we considered that the firing of one set of neurones (and so, the generation of a given class of CDP) depended on the last activated ensemble of neurones. That is, on the preceding CDP. In other words, we assumed that the generation of a given CDP could be described as a Markovian process of degree 1 (see Martin et al., 2017).

This means that the organization of the networks involved in the generation of the CDPs is not a simple probabilistic independent activation of the different ensembles of neurons, but rather a consequence of the structured connectivity between them. Hence, we assumed that the set of transition probabilities between the different classes of CDPs could provide relevant information on the state of functional connectivity between the different neuronal ensembles involved in the generation of the examined CDPs, both at rest and during the different experimental procedures. See Appendix for further details.

#### Likelihood Computation and Similarity Index Definition

To compare sequences of CDPs generated in the same segment during different experimental procedures, we defined a new similarity measure. First, each segment and time step was represented by a model consisting of the probability matrix of conditional dependence limited to the last CDP. This model was built from the sequence of CDPs recorded in segment *l*, at time step *s* under the assumption of a Markovian behavior of the sequence of order 1 (see Martin et al., 2017). We then computed the likelihood of this model to generate data recorded in another time step *s*’. This likelihood was used to estimate if different time steps obtained from the same lumbar segment under a specific experimental condition (e.g., control recordings) had or not a similar behavior. To build a similarity index for the different steps, instead of working with probability values, we worked (for numerical stability reasons) with the logarithm of the probabilities.

Finally, given that maximum likelihood for a given sequence obtained from the experimental data resembled the sequence from which the model was created, we normalized the log likelihood values to allow an easy comparison between steps. Values were within the range [0.1], with 0 when the probability of the model to generate a given sequence was 0, and 1 when the probability of generating the sequence was the same as that generated by the source data from which the model was built. Notice that this is not a symmetric measure. However, we still used it as an index of similarity to build neighborhood graphs, where non-symmetric relations are common.

#### Similarity Graph Generation

This approach was used to obtain a high-level interpretation of the behavior of the CDPs recorded in different spinal segments induced by several experimental procedures. To this end, for a given spinal segment, we computed the similarity index between CDPs recorded during successive time steps with a comparable Markovian structure. This information was visualized by constructing a neighborhood graph where each node represented a time step of the experiment and the edges connected steps that were considered similar using the criteria described in the previous section. In these graphs, nodes were connected only to the most similar nodes that also exceeded a threshold of similarity index. This procedure allowed display of only the highly significant connections. As detailed in the Appendix, the WalkTrap method (Pons and Latapy, 2005) was followed to identify clusters of nodes that share significant similarities.

#### Consensus Graph Generation

To obtain a general vision of the behavior of the whole lumbar ensemble of dorsal horn neurones generating the selected classes of CDPs, we generated a single consensus graph that included the information obtained from all segments. To this end, we applied ensemble methods used in machine learning that consider the expert’s predictions to obtain a single consensus graph. In order to increase the reliability of the obtained graphs we implemented a majority voting procedure to obtain a single representation of the whole behavior of the CDPs.

This procedure included the following: (a) given a lumbar segment, for each step in that segment we made a list with the most similar steps above a threshold. This list was considered as votes for the similar steps in that segment, (b) we collected the votes produced in all lumbar segments, (c) a consensus graph was built with a node for each step with lines joining the *k* most voted steps, and (d) the graph was segmented into clusters of nodes using the WalkTrap method. The consensus graph not only describes the overall behavior of the neuronal networks but also describes it with a degree or reliability higher than that obtained by observing the selected set of CDPs in each segment (see Appendix for more details).

#### Similitude Between Pairs of Histograms of CDPs

In order to assess the similarity in the probability distribution of the CDPs generated in a given segment during different maneuvers, we used a similarity measure to compare the histograms of the clusters of CDPs. This measure is based on the test developed in Bityukov et al. (2016). Let us consider a simple model with two histograms where the random variable in each bin obeys the normal distribution

(1) $$p\,\left(x|n_{i\,k}\right)=\frac{1}{\sqrt{2\,\pi \,\sigma_{i\,k}}}\,e^{-\frac{{\left(x-n_{i\,k}\right)}^{2}}{2\,\sigma_{i\,k}}}$$

where the expected value in the bin *i* is equal to *n*_*ik*_ and the variance $\sigma_{i\,k}^{2}=n_{i\,k}$ and *k* is the histogram number (*k* = 1, 2). We define the significance as

(2) $${\hat{S}}_{i}=\frac{{\hat{n}}_{i\,1}-{\hat{n}}_{i\,2}}{\sqrt{{\hat{\sigma }}_{i\,1}^{2}+{\hat{\sigma }}_{i\,2}^{2}}}$$

where ${\hat{n}}_{ik}$ is an observed value in the bin *i* of the histogram *k* and ${\hat{\sigma }}_{i\,k}^{2}$ = ${\hat{n}}_{ik}$. This model can be considered as the approximation of the Poisson distribution by the Normal distribution. The values *n*_*ik*_ (*i* = 1, 2,…, *M, k* = 1, 2) are the numbers of events appeared in the bin *i* for the histogram *k*. We consider the *RMS* (the root mean square) of the distribution of the significances

(3) $$R\,M\,S=\sqrt{\frac{\sum_{i=1}^{M}{\left({\hat{S}}_{i}-\bar{S}\right)}^{2}}{M}}$$

Here, *S̄* is the mean value of *Ŝ*_*i*_. The *RMS* measures the distance between two histograms. If total number of events *N*_1_ in the histogram 1 and total number of events *N*_2_ in the histogram 2 are different, then the normalized significance *Ŝ*_*i*_(*K*) is calculated as follows

(4) $${\hat{S}}_{i}\,\left(K\right)=\frac{{\hat{n}}_{i\,1}-K\,{\hat{n}}_{i\,2}}{\sqrt{{\hat{\sigma }}_{i\,1}^{2}+K^{2}\,{\hat{\sigma }}_{i\,2}^{2}}}$$

where *K* = *N*_1_*/N*_2_. The relation *RMS*^2^ = χ^2^*/M*− *S̄*^2^ exists for the distribution of significances where $\chi^{2}=\sum_{i=1}^{M}{\hat{S}}_{i}^{2}$. One can show that the distribution of observed significances is close to normal distribution with the *RMS* ∼ 1. This distance measure between two histograms has a clear interpretation: *RMS* ∼ 0 histograms are identical, *RMS* ∼ 1 both histograms are obtained from the same parent distribution, *RMS*≫ 1 histograms are obtained from different parent distributions.

## Results

### Effects of Capsaicin and Lidocaine on Different Classes of CDPs Recorded in Preparations With Intact Neuroaxis

**FIGURE 1:**
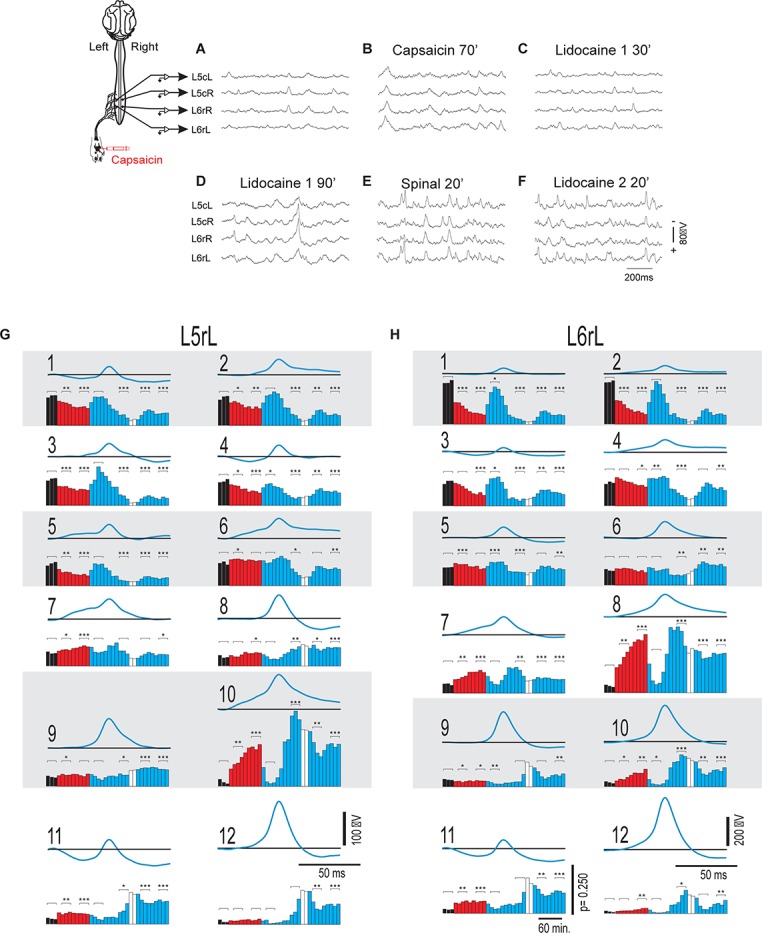
Changes in the shape dictionaries of the CDPs recorded in two spinal segments during the action of capsaicin and lidocaine and after spinalization. **(A–F)** Samples of raw recordings of the ongoing CDPs made in the left and right sides of the caudal region of the *L*5 segment (*L*5cL and *L*5cR) and rostral region of the *L*6 segment (*L*6rL and *L*6rR), as indicated. **(A)** Control. **(B)** 70 min after the intradermic injection of capsaicin in the left hindpaw. **(C,D)** 30 and 90 min after the first injection of lidocaine. **(E)** 20 min after a complete spinal transection at T4 and **(F)** 20 min after a second injection of lidocaine. **(G,H)** Shape dictionaries of the CDPs recorded in the *L*5rL and *L*6rL lumbar segments and histograms of the fractional probabilities of occurrence of each shape during successive time steps, as indicated. Black bars, control steps. Red bars, capsaicin steps; Blue bars, after the first administration of lidocaine. White bars, spinalization steps followed by a second administration of lidocaine. The brackets and asterisks over each set of columns indicate the statistical significance of the fractional probabilities of occurrence of each class of CDPs and the corresponding control probabilities (^∗∗∗^*p* < 0.001, ^∗∗^*p* < 0.01, and ^∗^*p* < 0.05). See text for further explanations.

As in previous studies (Manjarrez et al., 2000, 2003; Chávez et al., 2012; Contreras-Hernández et al., 2015; Martin et al., 2017), we found that the ongoing potentials recorded in the dorsum of the lumbar spinal segments included a series of brief potentials (CDPs), some of which appeared synchronously in different segments (Figure 1A). By 1 h after the nociceptive stimulation produced by the intradermal injection of capsaicin, the ongoing CDPs showed, in addition to the brief potentials, a series of slow synchronized oscillations (Figure 1B) that were transiently suppressed after the systemic injection of lidocaine, leaving sequences of brief potentials that resembled those recorded before the injection of capsaicin (Figure 1C). As the effect of lidocaine faded, the slow oscillations reappeared and were, in fact, more apparent than those recorded just before the administration of lidocaine (Figure 1D). Following acute spinalization, the slow synchronized oscillations were no longer observed and brief synchronized potentials were again generated (Figure 1E). These potentials appeared to be marginally affected by the second injection of lidocaine (Figure 1F).

One of the questions that were left unanswered in our previous study (Contreras-Hernández et al., 2018) pertained the mode of action of capsaicin and lidocaine on the patterns of functional connectivity between the dorsal horn neuronal ensembles involved in the generation of specific sets of CDPs. We assumed that some clues could be obtained by examining the changes on the probabilities of occurrence of the different classes of ongoing CDPs induced by nociception as well as during the antinociception produced by systemic lidocaine. To this end, we used machine learning (see section “Materials and Methods” and specially Martin et al., 2015) to identify and select the ongoing CDPs recorded in each segment according to their shape and amplitude and examine how capsaicin, lidocaine, and spinalization affected the fractional probabilities of occurrence of each class of CDPs.

Figures 1G,H show the means of the 12 different classes of CDPs selected from recordings made in segments *L*5rL (left rostral L5 lumbar segment) and *L*6rL (left rostral L6 lumbar segment). These CDPs were ordered according to their *control* probabilities of occurrence during each of the 10 min steps and displayed as vertical black bars in the corresponding histograms.

It may be seen that some of the selected CDPs started from a flat baseline and were purely negative, resembling the nCDPs reported in previous studies (e.g., classes 2 and 9 in *L*5rL and classes 1 and 6 in *L*6rL), while others were negative-positive (classes 8 and 12 in *L*5rL and classes 5, 9, and 12 in *L*6rL) resembling the npCDPs. There were in addition other classes of CDPs in which the main negative potential was preceded either by a slow negative component (classes 6, 7, and 10 in *L*5rL and classes 4, 7, and 8 in *L*6rL), or by a positive component (classes 1, 4, and 11 in *L*5rL and classes 3 and 11 in *L*6rL).

As shown by the black bars in the histograms, in both segments the fractional probabilities of occurrence of each class of the selected CDPs were relatively constant during the control periods (three consecutive 10 min bins in this case). Quite interestingly, the classes comprising the smallest CDPs recorded during the control periods had higher fractional probabilities of occurrence than the classes including the largest CDPs (see also Martin et al., 2015).

The red bars in the histograms show that the intradermal injection of capsaicin had mixed effects on the fractional probabilities of occurrence of the CDPs: they were gradually reduced in some of the classes comprising the smallest CDPs (classes 1–5 in segment *L*5rL and classes 1–3 in segment *L*6rL), increased in other classes of CDPs (classes 7, 8, and 10 in segment *L*5rL and classes 7, 8, 10–12 in segment *L*6rL), or else remained unaffected.

After the systemic injection of lidocaine (blue bars), the effects of capsaicin were reversed in a differential manner for a short time period (20–40 min). That is, lidocaine increased the probabilities of those CDPs showing low probabilities of occurrence after capsaicin and reduced the probabilities of the CDPs with higher probabilities. The reversal by lidocaine of the effects of capsaicin was over by 60–80 min after its systemic administration. After spinalization, the probabilities of occurrence of the different classes of CDPs (white bars) were also changed but to a smaller extent by a second injection of lidocaine.

We have performed a Student’s *t*-test to assess the changes in the fractional probabilities of occurrence in each of the different classes of CDPs displayed in the histograms of Figures 1G,H induced by capsaicin, lidocaine, and spinalization. To this end we used as reference the average of the three control probabilities that were compared with those obtained during the different procedures at the times indicated with the brackets on the histograms. Statistical significance between the different sets is indicated in the figure by the asterisks (^∗∗∗^*p* < 0.001, ^∗∗^*p* < 0.01, and ^∗^*p* < 0.05).

The finding that the shapes of the different classes of the CDPs selected in segment *L5*rL resembled rather closely those extracted from recordings made in segment *L6*rL (and in other lumbar segments as well) further supports our previous proposal that the different classes of CDPs are generated by the activation of a segmentally distributed ensemble of interconnected dorsal horn neurones (Manjarrez et al., 2000, 2003; Chávez et al., 2012; Contreras-Hernández et al., 2015; Martin et al., 2017).

### State Transitions

To have some information on the global state of the neuronal networks involved in the generation of the different classes of CDPs, we used similarity procedures to compare the probabilities of occurrence of all the classes of CDPs generated in a given segment during a particular moment with the probabilities of the potentials generated at another time in the same segment. For example, on the extent to which the set of CDPs recorded during the Control 1 period resembled the potentials recorded during the Control 2 period and so on.

**FIGURE 2:**
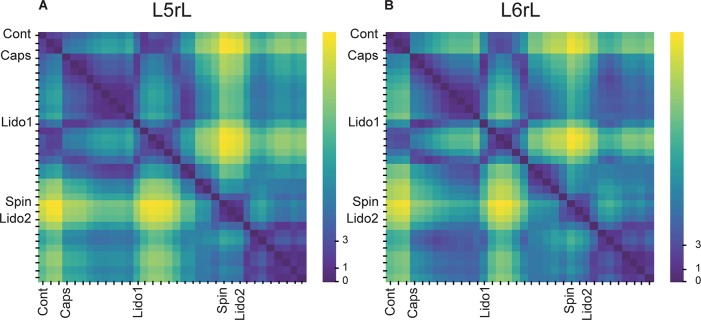
**(A,B)** Comparison of the histograms of probabilities of the CDPs recorded in two spinal segments (*L*5rL, *L*6rL) during the action of capsaicin and lidocaine and after spinalization using the RMS of the distribution of significances. Each change in maneuvre induces an important change in the distribution of the probabilities of occurrence of the CDPs with different shapes. Each matrix value shows RMS statistic for a given pair of steps of the experiment. Values closer to approximately 0–2 (darkest colors) mean that histograms of probabilities of CDPs in both steps are significantly similar according to RMS significance test. The higher the value of RMS, the higher the differences in the histogram of probability of the CDPs. See section “Similitude Between Pairs of Histograms of CDPs” for further details.

In order to assess the differences between the probabilities of occurrence of the whole set of the selected classes of CDPs during the different maneuvers we used a test based on the significance of differences between histograms (see section “Similitude Between Pairs of Histograms of CDPs”). The obtained RMS values were displayed in a matrix relating the similarity of the histograms. Figures 2A,B shows the evolution of the changes in the probabilities of occurrence of the CDPs recorded in two neighboring spinal segments (*L5*rL and *L6*rL), measured by the RMS of the distribution of significances.

This figure displays the RMS values between pairs of histograms. The lower the RMS values, the more similar the histograms (see color scale on the right). A zero RMS would indicate that both histograms were identical. Higher RMS values would mean increasing dissimilarities. It may be seen that both matrices were remarkably similar in general with minor differences in detail (see color patterns). For example, the first three vertical columns in the left side of the figure (control histograms) were rather similar to each other in both segments (low RMS values, dark blue). They became gradually dissimilar during the action of capsaicin, slightly more in the L6 than in the L5 segment (light blue, steps 1–3 to green, steps 4–9). The histograms obtained after the first injection of lidocaine became temporarily similar to the control histograms in both segments (shift to dark blue, steps 1–6) and dissimilar later on (shift to light green, steps 7–11), thus resembling the effects of capsaicin. Spinalization reduced similarity with control histograms rather abruptly (shift to yellow) that was again slightly increased after the second injection of lidocaine. Notice that the effects produced by lidocaine before and after spinalization were quite dissimilar.

**FIGURE 3:**
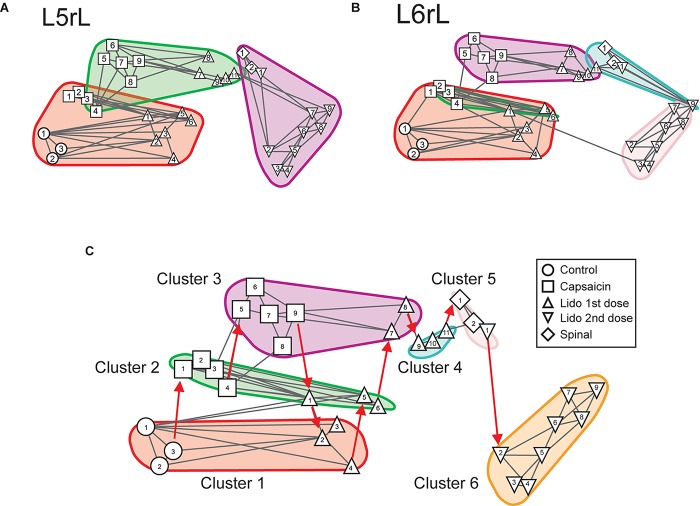
Effects of capsaicin, lidocaine, and spinalization on the similarity graphs of the CDPs recorded from different spinal segments. **(A,B)** Similarity graphs of the CDPs recorded in two different spinal segments, as indicated. These graphs were constructed with *k* = 3 and *S* = 0.9. Each node represents a different step of the experiment and each cluster includes nodes among the k most similar models found by applying the WalkTrap clustering method. **(C)** Consensus similarity graph generated with *k* = 4, *mst* = 0.9 and *k*^2^ = 3. Numbers in each node show the time step of the experimental procedure (see inset with symbols). Colors represent clusters of nodes grouped by similarity and numbers the successive steps. The red arrows in **(C)** indicate the temporal sequence of the transitions between the different nodes in each cluster. See text for further explanations.

Figures 3A,B illustrates the transitions produced by capsaicin, lidocaine, and spinalization on the different classes of CDPs extracted from the rostral regions of the *L5* and *L6* segments in the left side obtained from the same set of data as those used to generate Figure 1. Each graph shows the similarity of the whole set of CDPs obtained at a given moment with the CDPs recorded at other times. The structure of the CDPs recorded during each procedure can be identified by the internal similarities among each one of the steps as show in Figure 2. For example, in Figures 3A,B, control steps 1, 2, and 3 appear close together, both in segment *L*5rL and in segment *L*6rL, suggesting that the control distributions of the different classes of CDPs in each segment were rather similar and behaved alike in these segments. These control groups became clearly differentiated from the capsaicin steps 5–9 in segments *L*5rL and *L*6rL.

After lidocaine, the node distributions transiently resembled the control distributions (e.g., lidocaine steps 1–6 in *L*5rL and in *L*6rL). Later on (steps 7–11), the distributions attained in segments L5rL and L6rL resembled those seen during capsaicin steps 5–9. They became separated after spinalization (spinal steps 1 and 2). Quite interestingly, after the second injection of lidocaine applied in the already spinalized preparation, the distributions remained within the same cluster in segment *L*5rL but not in segment *L*6rL. The transitions between the different classes of CDPs produced by capsaicin and lidocaine illustrated in Figures 3A,B were not limited to the *L*5 and *L*6 segments, but were also seen in all the other spinal segments (not illustrated). Although each one of them displayed its own particular features, the transitions between the different classes of CDPs followed similar patterns, in the sense that in all segments each experimental procedure shifted the state of functional connectivity in a similar direction.

In other words, every group of steps from the individual graphs remained clustered nearly in the same way in all lumbar segments. So, if these structures were similar at the local level, it is possible that they would also do it in a global level.

### Consensus Graphs

We have used the consensus graphs to examine the effects of nociception and antinociception on the global behavior of the CDPs recorded in both sides in the *L*4 to *L*7 spinal segments. The data depicted in Figures 3A,B show state transitions of the CDPs generated in two spinal segments following the intradermal injection of capsaicin, how the systemic injection of lidocaine temporarily reversed these state transitions and how they were affected by a subsequent spinalization. It thus seemed of interest to examine the state transitions of the whole segmental ensemble of CDPs induced by the different experimental procedures. That is, of the global state transitions. This was achieved by building a unique consensus graph using a majority voting procedure (see section “Materials and Methods” and Appendix).

Figure 3C shows the consensus graph obtained from the data recorded in segments L4–L7 in both sides, in the same experiment as that of Figure 1. In this case, similar nodes were grouped in the same cluster. The red arrows indicate the temporal sequence of the transitions between the different nodes in each cluster induced by capsaicin, lidocaine, and spinalization.

Cluster 1 includes the control nodes (control steps 1–3, circles). The injection of capsaicin produced an initial transition of the nodes to cluster 2 (capsaicin steps 1–4, squares), and later on to cluster 3 (capsaicin steps 5–9, squares). The injection of lidocaine again shifted the nodes, initially to cluster 2 (lidocaine step 1, upward triangle) and later on to cluster 1 (lidocaine steps 2–4, upward triangles) resembling the control nodes.

As the effect of lidocaine faded, the nodes appeared in cluster 2 (lidocaine steps 5–6, upward triangles), later on in cluster 3 (lidocaine steps 7–8, upward triangles) and then in cluster 4 (lidocaine steps 9–11, upward triangles). Clusters 5 and 6 include the nodes obtained after spinalization (diamonds) and the second injection of lidocaine (downward triangles), respectively. It is quite clear that the configuration of the CDPs in these clusters had no resemblance with the configuration seen before spinalization.

**FIGURE 4:**
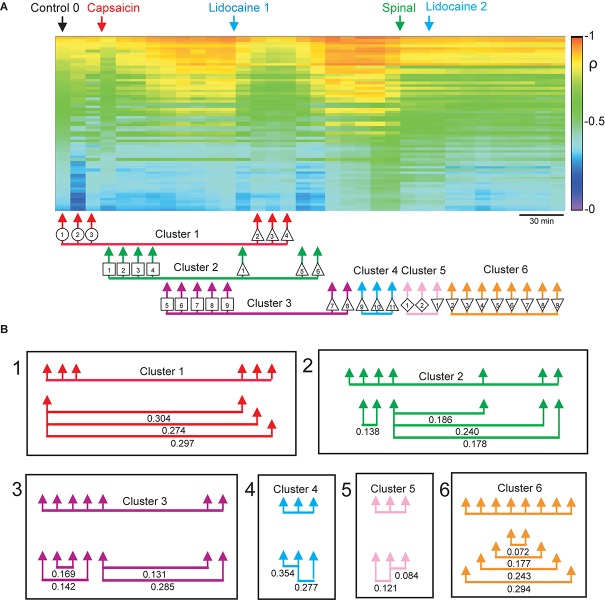
Changes in correlation and similarity between paired sets of CDPs produced by capsaicin, lidocaine, and spinalization. **(A)** Coefficients of correlation between paired sets of CDPs generated during successive 10 min recording periods. The coefficients of correlation obtained during the Control 0 period are shown in descending order as a vertical column. The coefficients of correlation of recordings made at subsequent times are displayed keeping the same order as the Control 0 coefficients. Colors show the magnitude of the correlation (see scale). Arrows show time of capsaicin and lidocaine injections and of spinalization. The arrows joined with a horizontal line below the correlogram comprise the nodes with similar classes of CDPs shown in the consensus graph of Figure 3C. **(B)** Similarity indices between the sets of coefficients of correlation included in each of the clusters shown in the consensus graph. Note that the arrangement of the clusters and nodes obtained from the consensus graphs after the different experimental procedures resembles rather closely the concurrent changes in the correlation between paired sets of CDPs, as indicated by the similarity indices. Further explanations in the text. The graph displayed in A was taken from Contreras-Hernández et al. (2018) and is reproduced with permission of *Journal of Physiology*.

In Contreras-Hernández et al. (2018), we examined the functional relations between the dorsal horn neuronal networks involved in the generation of the synchronized activity in different spinal segments by calculating the coefficients of correlation between the different sets of segmental potentials recorded during 10 min periods. These data were used to construct the correlogram illustrated in Figure 4A. The first column in this correlogram (Control 0) displays the coefficients of correlation between the different paired sets of *L*4–*L*7 CDPs recorded with the ensemble of 12 electrodes. These coefficients were arranged in decreasing order and displayed vertically (see colored scale). The other columns show the coefficients obtained during successive 10 min non-overlapping recording periods made continuously along the whole experiment. They were displayed keeping the same order as that of the coefficients obtained during the control 0 recording period.

It may be seen that capsaicin gradually increased the correlation between the CDPs recorded from different segments and that this effect was transiently reduced after the systemic injection of lidocaine. The patterns in correlation observed after the effects of lidocaine faded were drastically changed after a high acute spinalization and were barely affected by the second injection of lidocaine (for more details see Contreras-Hernández et al., 2018).

This graph was introduced to compare the changes in the coefficients of correlation between the CDPs induced by capsaicin, lidocaine, and spinalization with the state transitions inferred from the consensus graph displayed in Figure 3C. The arrows and symbols together with the horizontal lines at the bottom of the correlogram in Figure 4A show the distribution of the six clusters and their nodes obtained from the consensus graph illustrated in Figure 3C. The nodes belonging to the same cluster are joined with a horizontal line.

As shown in Figure 4B, there was a clear correspondence between the grouping of the nodes in particular clusters and the patterns of correlation between the CDPs displayed during the different periods. This correspondence was assessed by comparing the similarity indices between the sets of coefficients of correlation indicated by the nodes obtained from the consensus graphs illustrated in Figure 3C. A similarity index of 0 would indicate identity between the two sets of coefficients of correlation, while an index of 1.0 would indicate that the two sets were completely different (see section “Materials and Methods”).

It then follows that the data sets included within cluster 1 had similarity indices between 0.274 and 0.304, suggesting a reasonable similarity between them. This was also the case for the data included in clusters 2 and 3, as well as for clusters 4–6. It should be noted that after spinalization, the similarity indices of the data included in clusters 5 and 6 were pretty low, suggesting that in the absence of descending influences, the correlation between the CDPs recorded from different segments was relatively steady, even after the second injection of lidocaine (see Contreras-Hernández et al., 2018, for further details).

**FIGURE 5:**
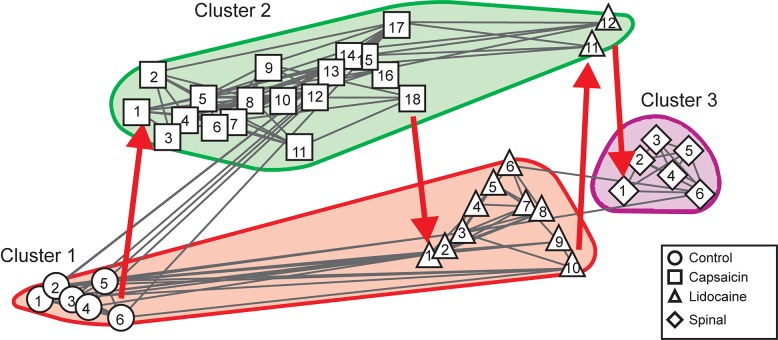
Consensus similarity graph obtained in a preparation with intact neuroaxis. Cluster 1 included six similar control nodes. By 10 min after the injection of capsahanges in the patterns of simicin they shifted to Cluster 2, where they stayed during 18 consecutive time steps. Immediately after the injection of lidocaine the nodes shifted back to cluster 1, remained there during 10 steps and moved back to cluster 2 where they stayed during two additional steps. After spinalization, the nodes shifted to cluster 3 and stayed there for at least six additional steps. This consensus graph was constructed with *k* = 4, *mst* = 0.9, and *k*^2^ = 4. The red arrows indicate the temporal sequence of the transitions between the different nodes in each cluster. See text for further details.

Figure 5 shows the consensus graph obtained in another experiment where we followed the same protocol. In this case, the nodes were grouped in three clusters. Cluster 1 comprised six similar control nodes (steps 1–6, circles). By 10 min after the injection of capsaicin, the nodes shifted to cluster 2 where they remained during 3 h (steps 1–18, squares). Immediately after the injection of lidocaine (step 1, upward triangles) the node shifted again to cluster 1 and so were the nine following steps (steps 2–10, upward triangles). Later on, lidocaine steps 11 and 12 shifted back to capsaicin cluster 2. After spinalization, the nodes were again shifted, this time to a separate cluster and remained there for at least 1 h (spinal steps 1–6).

In summary, the consensus graphs depicted in Figures 3C, 5 indicate that capsaicin changes the state of functional connectivity between the neurones involved in the generation of the different classes of CDPs and that the acquired state is temporarily reverted to the control configuration by lidocaine. They also show that the transition between clusters produced by capsaicin and lidocaine is shaped by supraspinal influences that are suppressed after spinalization.

### Which Are the Effects of Capsaicin and Lidocaine Applied in Previously Spinalized Preparations?

In Contreras-Hernández et al. (2018), we showed that after spinalization the effects of capsaicin and lidocaine on the correlation between different sets of CDPs were significantly attenuated. Yet, the question remained if capsaicin and lidocaine had some action when applied in previously spinalized preparations. Clearly these two situations are not necessarily equivalent, because capsaicin applied in a preparation with intact neuroaxis will activate ascending pathways reaching supraspinal structures which in turn promote descending influences that modulate the functional connectivity between the dorsal horn neuronal ensembles distributed along the lumbar segments (Ramírez-Morales et al., 2019). These descending control mechanisms include activation of 5-HT and dopaminergic fibers that may produce long lasting changes of synaptic transmission along a diversity of spinal pathways, including activation of silent synapses (Suzuki et al., 2004). Application of capsaicin in a previously spinalized preparation would certainly eliminate the descending control activated by the nociceptive stimulus.

**FIGURE 6:**
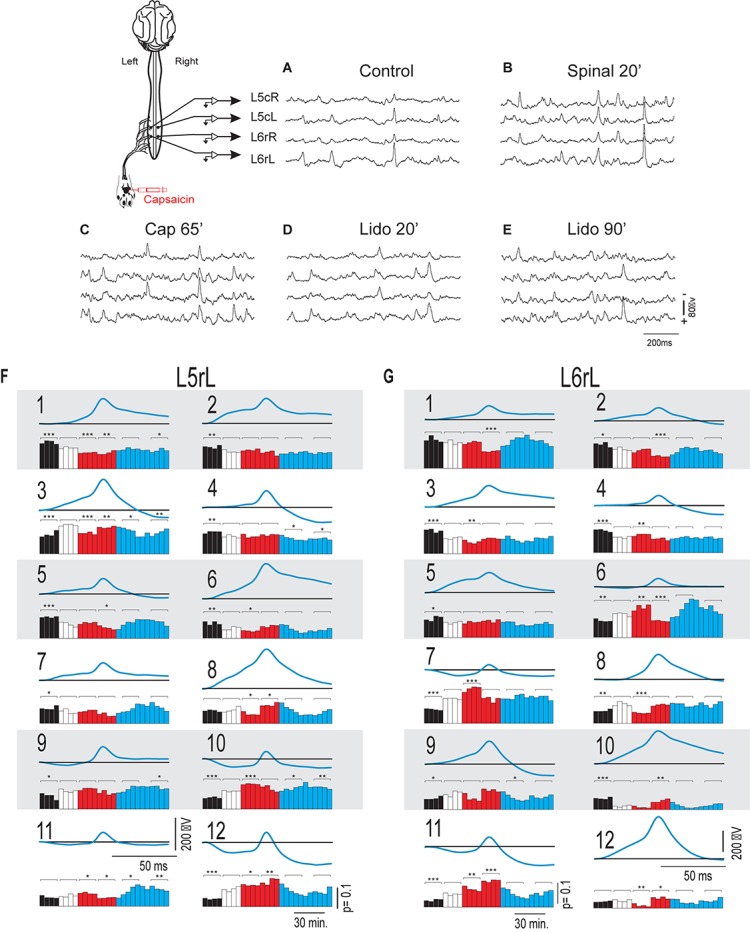
Changes in the shape dictionaries of the CDPs during the action of capsaicin and lidocaine injected in a previously spinalized preparation. **(A–E)** Spontaneous CDPs recorded in the left and right sides of the caudal region of the *L*5 segment and rostral region of the L6 segments, as indicated. **(A)** Control. **(B)** 20 min after spinalization. **(C)** 65 min after the intradermic injection of capsaicin in the left hindpaw. **(D,E)** 20 and 90 min after the systemic injection of lidocaine. **(F,G)** Shape dictionaries of the CDPs recorded in the *L*5rL and *L*6rL segments and histograms of fractional probabilities of occurrence for each shape at different time steps as indicated. The brackets and asterisks over the columns indicate the statistical significance of the fractional probabilities of occurrence of each class of CDPs relative to the fractional probabilities attained just after spinalization (^∗∗∗^*p* < 0.001, ^∗∗^*p* < 0.01, and ^∗^*p* < 0.05). See text for further explanations.

Figures 6A–E shows the ongoing CDPs recorded in the *L*5 and *L*6 segments in both sides of the spinal cord and how this activity was changed by spinalization and by the subsequent injection of capsaicin and lidocaine. It may be seen that spinalization increased the frequency of the ongoing CDPs, some of which remained synchronized (Figures 6A,B). The records displayed in Figure 6C were taken 65 min after the intradermal injection of capsaicin and resembled those displayed in Figure 6B, suggesting that capsaicin had a relatively weak effect on the functional relations between the neuronal sets involved in the generation of the different classes of CDPs. Nevertheless, the low frequency activity recorded after the injection of capsaicin was temporarily reduced following the systemic injection of lidocaine (Figures 6D,E).

Figures 6F,G shows the 12 classes of CDPs that were selected with the machine learning procedures from recordings made in the *L*5rL and *L*6rL segments, respectively. These classes were obtained from recordings made before spinalization and were used as a reference for the selection of the different classes of CDPs generated after spinalization as well as after the administration of capsaicin and lidocaine. In general, the selected CDPs resembled those observed in the preparation with intact neuroaxis displayed in Figures 1G,H. That is, some classes of CDPs started from a flat baseline and were negative or negative positive, while slow negative or positive potentials preceded other classes of CDPs.

The histograms displayed below the CDPs show that spinalization slightly increased the probability of occurrence of some of these potentials (e.g., classes 3, 9, 10, and 12 in segment *L*5rL and classes 6, 7, 8, and 11 in segment *L*6rL, while at the same time the probabilities of other classes were reduced (classes 1, 2, 4, 5, and 6, in *L5*rL and classes 3, 4, and 5 in *L*6rL), or else remained unchanged.

In contrast with what has been observed in the preparation with intact neuroaxis (Figures 1G,H), capsaicin injected after spinalization had rather small effects on the fractional probabilities of occurrence of the CDPs. Some were weakly and transiently increased (classes 5 and 10 in *L*5rL and classes 6, 7, and 11 in *L*6rL) while others were slightly reduced (classes 1 and 3 in *L*5rL and classes 8, 9, and 10 in *L*6rL). The statistical significance of the changes produced by capsaicin and lidocaine on the fractional probabilities of occurrence of the different classes of CDPs relative to the probabilities of the potentials recorded after spinalization (white columns) is indicated by the brackets and asterisks above the corresponding columns.

Another difference with the changes of the CDPs observed in the preparation with intact neuroaxis was that in the spinalized preparation the capsaicin-induced changes had a slower time course. Lidocaine applied after capsaicin also increased the probabilities of occurrence of some classes of CDPs and reduced the probabilities of other classes, but the effects were relatively small.

**FIGURE 7:**
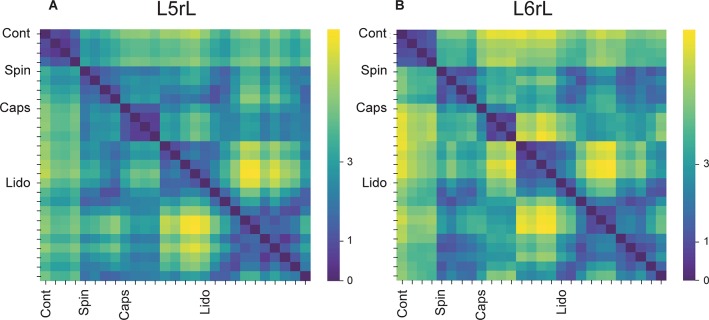
**(A,B)** Comparison of the histograms of probabilities of the CDPs recorded in two spinal segments (*L5*rL, *L6*rL) during the action of capsaicin and lidocaine in a previously spinalized preparation. Same format as that of Figure 2. Note that immediately after spinalization the patterns of the ongoing CDPs were drastically changed and were quite different from the control patterns (shift from deep blue to green). Yet, they were still modified following the administration of capsaicin and lidocaine suggesting persistence of dynamic processes. See text for further explanations.

**FIGURE 8:**
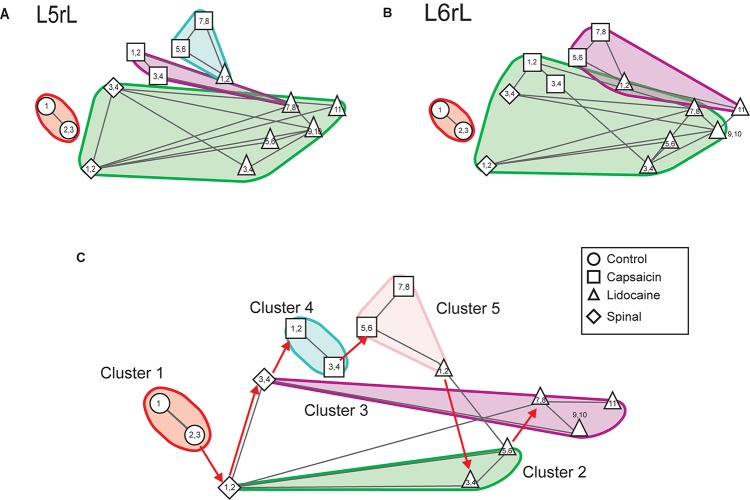
Effects of capsaicin and lidocaine applied after spinalization on the similarity and consensus graphs of the CDPs recorded from different spinal segments. **(A,B)** Similarity graphs of CDPs recorded in two different spinal segments, as indicated. These graphs were constructed with *k* = 3 and *S* = 0.9. As in Figures 3A,B, each node represents a different step of the experiment and each cluster includes nodes among the k most similar models found by applying the WalkTrap clustering method. **(C)** Consensus similarity graph (*k* = 4, *mst* = 0.9, *k*^2^ = 3). See text for further explanations.

Figures 7A,B show the changes in the patterns of similarity between the different sets of CDP histograms. It may be seen that after spinalization the histograms representing the global behavior of the CDPs recorded in the *L5*rL and *L6*rL segments showed no similarity with the histograms obtained before the spinal section. Yet, capsaicin and lidocaine changed the similarity patterns, apparently in the same direction as in the preparation with intact neuraxis. These changes lead to the grouping of the histograms in separate clusters (Figures 8A,B).

Figure 8C shows the consensus graph of the effects produced by capsaicin and lidocaine applied after spinalization. These graphs were obtained from the data recorded in all segments in the same experiment as that of Figure 6. In the consensus graph, similar nodes were grouped in five clusters. The red arrows indicate the temporal sequence of the transitions between the different clusters induced by spinalization, capsaicin, and lidocaine. It may be seen that spinalization first shifted the control nodes (control steps 1–3, circles) from cluster 1 to cluster 2 (spinal steps 1–2, diamonds) and later on to cluster 3 (spinal steps 3–4, diamonds). The injection of capsaicin shifted the nodes to cluster 4 (capsaicin steps 1–4, squares) and later on to cluster 5 (capsaicin steps 5–8, squares) that also included the CDPs recorded during the first 20 min after the injection of lidocaine (lidocaine steps 1–2, upward triangles). After that, the nodes first shifted to cluster 2 (lidocaine steps 3–6, upward triangles) and later on to cluster 3 where they remained until the end of the recording period (lidocaine steps 7–11, upward triangles).

**FIGURE 9:**
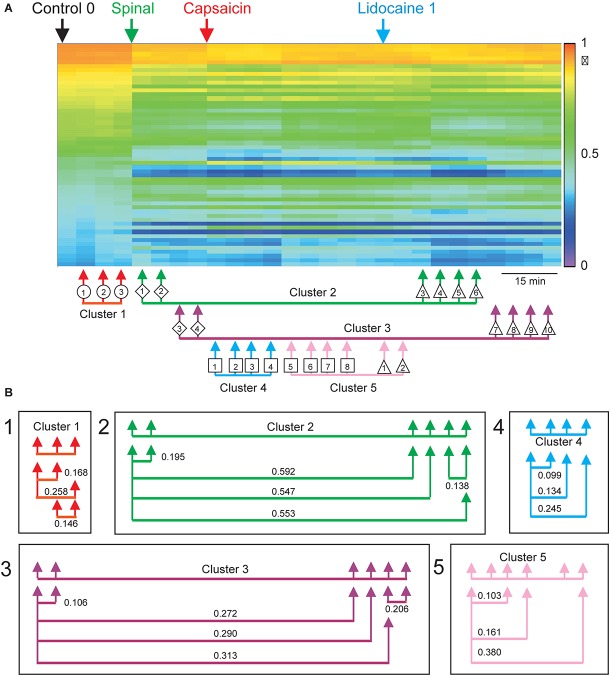
Effects of capsaicin and lidocaine applied after spinalization on the correlation between the paired sets of CDPs. Same format as that of Figure 4. **(A)** Changes in correlation between the different paired sets of CDPs produced by spinalization, capsaicin, and lidocaine. The arrows joined with an horizontal line displayed below the correlogram comprise nodes with similar classes of CDPs taken from the consensus graph shown in Figure 8C. **(B)** Similarity indices between the sets of coefficients of correlation during some of the steps obtained from the consensus graph, as indicated. Further explanations in text. The graph displayed in A was taken from Contreras-Hernández et al. (2018) and is reproduced with permission of *Journal of Physiology*.

The correlogram depicted in Figure 9A allows comparison of the changes in the patterns of correlation between the CDPs induced by capsaicin and lidocaine in the already spinalized preparation with the distribution of the nodes and clusters obtained from the consensus graphs displayed in Figure 8C. It may be seen that in the absence of a supraspinal control, capsaicin and lidocaine still affected the functional connectivity between the dorsal horn neurones, even though the changes in correlation between the CDPs recorded from different segments were not as marked as those seen in preparations with intact neuroaxis (see Contreras-Hernández et al., 2018, for further details).

**FIGURE 10:**
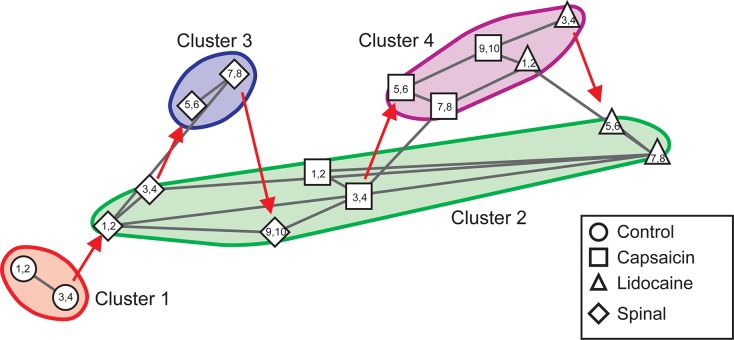
Consensus similarity graph obtained in another experiment in which capsaicin and lidocaine were administered after acute spinalization. The nodes of the CDPs recorded before spinalization were all grouped in cluster 1. Those obtained soon after spinalization were transiently shifted to cluster 2, then to cluster 3 and later on back to cluster 2, that also comprised the nodes obtained during the first 4 time step after the injection of capsaicin. Subsequently, the nodes shifted to cluster 4 where they remained during six time steps. By 4 time steps after the injection of lidocaine they shifted back to cluster 2. This consensus graph was constructed with *k* = 4, *mst* = 0.9, and *k*^2^ = 3. The red arrows indicate the temporal sequence of the transitions between the different nodes in each cluster. See text for further details.

Figure 10 displays the consensus graph constructed with data obtained in another experiment. It may be seen that the nodes of the segmental CDPs recorded before spinalization (steps 1–4, circles) were rather similar and were grouped in cluster 1. Those obtained soon after spinalization (steps 1–4, diamonds) were transiently shifted to cluster 2, then to cluster 3 (steps 5–8, diamonds) and back to cluster 2 (steps 9–10, diamonds), that also comprised the nodes obtained during the first 40 min after the injection of capsaicin (steps 1–4, squares). Subsequently, the nodes shifted to cluster 4 (steps 5–10, squares) that also included the nodes generated during the first 40 min after lidocaine (steps 1–4, upward triangles) and finally to cluster 2 (steps 5–8, upward triangles).

In summary, the consensus graphs depicted in Figures 8C, 10 indicate that in the previously spinalized preparation, capsaicin, and lidocaine also change the state of functional connectivity between the neurones involved in the generation of the different classes of CDPs. Yet, as shown in Figure 9A (see also Contreras-Hernández et al., 2018), in the spinal preparation the effects on the overall correlation between the CDPs produced by capsaicin and lidocaine were relatively weak, suggesting that in the preparation with intact neuroaxis the transitions between clusters during nociception and antinociception is dominated by supraspinal influences. At this point, we have no information on whether in the spinal preparation the same or different dorsal horn neurones are activated by capsaicin before or after spinalization and on the kind of interaction that these neurones have with other spinal pathways.

## Discussion

### Capsaicin and Lidocaine Change in a Structured Manner the CDPs Selected With Machine Learning Procedures

We have now used machine learning similarity procedures to build dictionaries of spontaneous CDPs selected according to their shape and amplitude to disclose the changes produced in each of them by the experimental procedures associated with nociception (see Martin et al., 2015). We found that under control conditions (i.e., before capsaicin, lidocaine, or spinalization) most of the classes comprising the smallest CDPs were produced during a state of low neuronal synchronization. They occurred more often than classes comprising the largest CDPs that were instead generated during states of higher synchronization. We also found that capsaicin had opposite effects on the fractional probabilities of occurrence of some classes comprising the smallest and largest CDPs.

The differential action of capsaicin on the different classes of CDPs could be explained assuming that these population potentials were generated by a segmentally distributed ensemble of interconnected dorsal horn neurones, and that the changes in the probabilities of occurrence of each class resulted from dynamic modifications in the interaction between neurones within the same ensemble, as it has been proposed for the generation of the nCDPs and npCDPs (Contreras-Hernández et al., 2015).

An alternate explanation to the differential action of capsaicin in preparations with intact neuroaxis would be that each class of CDPs was generated by a specific set of strongly interconnected neurones (modules?) distributed along the different spinal segments. Capsaicin would inhibit, either directly, or via descending pathways, the neurones in some of the modules and at the same time activate the neurones in other modules, perhaps via the on and off brain-stem neurones (Basbaum and Fields, 1978; Urban and Gebhart, 1999; Vanegas and Schaible, 2004; Brooks and Tracey, 2005; Smith et al., 2006; Ossipov et al., 2010; Brink et al., 2012).

It should be noted that a fair number of the selected CDPs were preceded by slow negative or positive potentials and few started from a flat baseline (see Figures 1G,H, 6F,G). At present we don’t know if these potentials were produced by the same neuronal ensemble that generates the selected CDPs or if they were generated by the activation of separate neuronal ensembles that affected the probabilities of occurrence of the ensembles involved in the generation of the CDPs, as suggested by Markovian analysis (see below).

The finding of some classes of CDPs whose probabilities of occurrence were not changed by capsaicin and lidocaine suggests in addition that the neurones involved in the generation of these potentials were not directly related to nociception but could still control information transmission in other pathways as suggested by recent observations of Ramírez-Morales et al. (2019), who found that the dorsal horn field potentials produced by activation of low threshold afferents signaling joint position, were basically unaffected after the intradermic injection of capsaicin, in contrast with the facilitation of the responses produced by stimulation of high threshold myelinated fibers conveying nociceptive information.

Future research should be addressed to relate the activity of individual, functionally characterized dorsal horn neurones with the different classes of CDPs and to examine how this relation is affected during nociception and antinociception.

### The Assemblage of CDPs in a Specific Cluster Defines the State of Functional Connectivity Between Specific Sets of Dorsal Horn Neurones

By using a Markovian approach, we were able to compare the similarities between the whole set of CDPs recorded in a given segment with the CDPs generated in the same segment under different experimental conditions (Figures 3A,B). We assumed that grouping in the same cluster the different classes of CDPs according to similarity criteria defines the functional state of connectivity of the dorsal horn neuronal network in that particular segment at a given moment. In contrast, the uniquely generated consensus graphs (see Figure 3C) provide a general vision of the behavior of the whole ensemble of dorsal horn neurones by including information obtained from all segments with a degree of reliability higher than that obtained by observing the selected sets of CDPs in each segment. The clusters displayed in the consensus graphs would then illustrate the transition between the different functional states induced by nociception and antinociception.

Our observations suggest further that the system does not operate as a causally deterministic relay but instead as a probability system able to process and/or modulate the output behavior (increased/decreased/total blockade) through the operation of specific sets of intraspinal neurones. It is tempting to suggest that the spontaneous synchronous and coordinated neuronal activity recorded in the dorsal horn of the spinal cord represents pieces of complex dynamic adaptive behaviors associated to particular functional states of the spinal neuronal networks that may behave as functional units that control the balance between excitation and inhibition, as part of the homeostatic processes involved in health and disease. It thus seems possible, as suggested in our previous study (see Contreras-Hernández et al., 2018), that the changes in spinal circuitry produced by acute nociceptive stimulation are part of the response of the system in conditions of self-organized criticality in which descending control can shift the spinal neuronal networks to a different functional state.

In this context, we would like to point out that even though the term “adaptation” has been traditionally used to describe the reduction of the responses recorded in afferent fibers following sustained activation of peripheral receptors (Adrian and Zotterman, 1926), as well as the modifications of the reflex responses produced by repeated sensory stimulation (Grau et al., 2014), a more general definition of adaptation comprises the frequently used term “adaptive” that characterizes dynamic changes in neuronal connectivity under a variety of physiological and pharmacological conditions used to induce contextual modulations in neuronal functional connectivity lasting for minutes that shape transmission of sensory information in a structured manner (Holland, 2006).

The exact nature and mechanisms of criticality, and its functional role are still an open question. Criticality is a fundamental concept to understand the operation of complex adaptive systems and is defined as a specific type of behavior observed when a system undergoes a transition between different phases (Strogatz, 2000). Criticality maximizes the dynamic range of the responses to different inputs (Kinouchi and Copelli, 2006; Shew et al., 2009, 2011; Yang et al., 2012; Meisel et al., 2013), and it has been proposed that complex dynamical adaptive systems such as large neuronal networks in the central nervous system operate in a critical state through an active decentralized process known as self-organized criticality (Bak et al., 1988; Bornholdt and Rohlf, 2000; Levina et al., 2009; Meisel and Gross, 2009; Droste et al., 2013). One possible explanation to the differential action of capsaicin on the functional connectivity (correlation) between the dorsal horn neuronal networks involved in the generation of the different classes of CDPs, as well as on their different probabilities of occurrence, might be related to different states of criticality induced by nociception simultaneously on different neuronal ensembles.

### Some Functional Implications of the Capsaicin-Induced Changes on the Probabilities of Occurrence of the Different Classes of CDPs

We have shown previously that the spontaneous nCDPs recorded in the lumbar cord were associated with states of low neuronal synchronization and that in these conditions there was a concurrent activation of the pathways mediating non-reciprocal Ib post-synaptic inhibition. In contrast, during states of increased neuronal synchronization, as well as after the acute section of the superficial peroneal (SP) and sural (SU) nerves, npCDPs were preferentially generated, suggesting increased activation of the pathways leading to primary afferent depolarization and presynaptic inhibition (Chávez et al., 2012; Contreras-Hernández et al., 2015).

These findings led us to examine the extent to which the nociceptive stimulation produced by the intradermal injection of capsaicin in preparations with intact neuroaxis affected the probabilities of occurrence of the CDPs selected with the machine learning procedures, even though so far the nCDPs and npCDPs are the only classes of CDPs that we have been able to associate with the activation of specific inhibitory pathways.

The data displayed in Figures 1G,H show that capsaicin had a differential action on the fractional probabilities of occurrence on the different classes of CDPs. We have assumed that these changes contribute to the development of the capsaicin-induced hyperalgesia. Although the individual and global contribution of each class to the development of hyperalgesia remains as an open question, it is tempting to suggest that the decreased probabilities of occurrence of the smallest CDPs (most of them nCDPs) led to a reduced activation of glycinergic neurones and this effect had some role in the development of hyperalgesia and allodynia, as suggested by the observations of Foster et al. (2015) who showed that the targeted ablation or silencing of glycinergic dorsal horn neurones induces localized mechanical, heath and cold hyperalgesia. It should be noted that there were other negative CDPs whose fractional probabilities of occurrence were instead increased by capsaicin (e.g., classes 7 and 10 in *L*5rL and classes 7, 8, and 10 in *L*6rL; Figures 1G,H) whose role in the activation of inhibitory pathways is still unknown.

In the experiment of Figure 1, the capsaicin-induced changes on the npCDPs were rather small, suggesting that activation of the pathways mediating PAD and presynaptic inhibition was not as strong as expected. However, in other experiments (see Martin et al., 2015), in addition to the capsaicin-induced reduction of the probabilities of occurrence of the classes comprising the smallest nCDPs, the probabilities of occurrence of some npCDPs were clearly increased, just as it was observed following the acute section of a cutaneous nerve (see Chávez et al., 2012), although it should be considered that these two procedures (capsaicin and nerve section) are not necessarily equivalent.

It thus seems possible that the scarce effects of capsaicin on the probabilities of generation of npCDPs in the experiment illustrated in Figures 1G,H were determined by the state of functional neuronal connectivity exhibited by the ensemble at the time of the intradermic injection of capsaicin (see Contreras-Hernández et al., 2018) which may to some extent depend on anesthesia depth as well as on the magnitude of the descending inhibitory control incremented by nociceptive stimulation (see Ramírez-Morales et al., 2019).

## Conclusion

The present set of observations was performed to further disclose the changes in functional connectivity between the segmental populations of dorsal horn neurones under conditions of nociception-antinociception and to evaluate the extent to which these changes were shaped by supraspinal influences.

The method presently employed goes beyond the measurement of global changes in correlation between the activity recorded from different spinal segments (Contreras-Hernández et al., 2018). It focuses on the changes in the fractional probabilities of occurrence of specific classes of CDPs selected by machine learning according to their shape and amplitude.

Our observations provide evidence of an intrinsic organization of the neuronal ensembles generating the different classes of CDPs as well as of the participation of descending influences in this organization during nociception and antinociception. It is tempting to speculate that the interaction between subpopulations of neurones through changes in the frequency of the CDPs (probabilities) observed under different experimental conditions represents the activation of specific sets of strongly interconnected sets of neurones acting as biological switches that address nociceptive information flow to particular pathways in the spinal cord and also to supraspinal structures.

Our present findings should not be taken as an assertion that spinal neuronal circuitry devoid of supraspinal control is unable to change its patterns of functional connectivity and their interaction with other spinal pathways in response to nociception and antinociception. In fact, as shown in Figures 7–10, in the previously spinalized preparation, the transition and consensus graphs representing population neuronal activity, do change after the administration of capsaicin and lidocaine. This situation could well-underlie the plasticity and learning of the spinal circuitry in response of nociceptive stimulation demonstrated by Grau et al. (2014) in chronically spinalized rats. It will be interesting to investigate if this form of plasticity can be also demonstrated in the acute preparation using the methodology presently described.

The effect of systemic lidocaine is remarkable in the sense that this local anesthetic counteracts the plastic neuronal changes induced by capsaicin. One possibility would be that lidocaine temporarily *erases* the state of central sensitization developed in a variety of supraspinal structures (i.e., brainstem, hippocampal and thalamocortical networks; see Drdla-Schutting et al., 2012; Bonin and De Koninck, 2014). Another possibility, suggested by recent observations (Plamenov et al., 2018) would be that lidocaine decorrelates and/or decouples the information flowing from the brainstem nuclei to the spinal circuitry, without significantly affecting the process of central sensitization that persists at higher levels.

We wondered on the extent to which the changes produced by capsaicin and lidocaine disclosed by the consensus graphs had a functional meaning, in the sense that they indeed represented different states of functional connectivity between the different ensembles of dorsal horn neurones at rest and after nociceptive stimulation and antinociception, or if they were computational constructions made without considering the context in which each of the specific classes of CDPs were generated. That is, of the state of functional connectivity displayed by the neuronal ensembles in the lumbar segments at that moment. In our view, the close resemblance between the arrangements of the clusters and nodes obtained from the consensus graphs and the concurrent changes in the patterns of global correlation induced after the administration of capsaicin and lidocaine illustrated in Figures 4, 9 validates the use of the consensus graphs as indicators of global changes in functional state observed during nociception and antinociception. It provides a tool to examine the changes in the functional role of the different populations of dorsal horn neurones that generate the spontaneous CDPs during different physiological and pathological conditions.

## Significance

We used Machine Learning and Markovian methods to examine the effects of nociception and antinociception analgesia on specific classes of the ongoing cord dorsum potentials (CDPs) generated in the lumbar segments of the anesthetized cat. We found that in preparations with intact neuroaxis, the machine learning selected classes of CDPs displayed structured (non-random) configurations that were changed by the intradermic injection of capsaicin to other, also non-random configurations. The systemic injection of lidocaine, a procedure known to decrease the manifestations of neuropathic pain, transiently reversed these configurations to their pre-capsaicin structure. It is suggested that the dorsal horn neuronal networks involved in the generation of the different classes of CDPs operate in a state of self-organized criticality as part of the homeostatic processes shaped by supraspinal descending influences in response to nociception.

## Data Availability

The datasets generated for this study are available on request to the corresponding author.

## Ethics Statement

Cats were bred and housed under veterinary supervision at the Centro de Investigación y de Estudios Avanzados of the Instituto Politécnico Nacional Animal Care unit (SAGARPA permission AUT-B-C-0114- 007). All experiments were approved by the Institutional Ethics Committee for Animal Research (protocol no. 126-03) and of the National Institutes of Health (Bethesda, MD, United States; Animal Welfare Assurance No. A5036-01). The Guide for the Care and Use of Laboratory Animals (National Research Council, 2010) was followed in all cases.

## Author Contributions

PR, SG, DC, and EC-H conceived and designed the experiments. DC, EH, EC-H, SG, and PR conducted the experiments. DC, EC-H, SG, and PR collected and interpreted the data. MM, JB, UC, AR-M, and LM programmed and analyzed the data. PR, SG, MM, BJ, and UC drafted the manuscript and reviewed it critically for important intellectual content. MM, JB, and UC performed the machine learning and the Markovian analysis of the experimental data. All authors have approved the final version of the manuscript and agreed to be accountable for all the aspects of the work.

## Conflict of Interest Statement

The authors declare that the research was conducted in the absence of any commercial or financial relationships that could be construed as a potential conflict of interest.

## Appendix

### Extraction of CDPs

The first step aimed to build dictionaries of events that were relevant for the analysis. In our domain, these events were the CDPs. As the initial step, an automated and unsupervised CDP detection method was applied. This method assumes smoothness in the definition of the CDP candidates and considers that they appear as peaks in the signal. We have assumed that the noise in the background of the recordings is stationary and Gaussian, and also independent of the neuronal signal. Given these assumptions, the signal was subjected to an automatic event extraction algorithm that detects peaks. This algorithm uses a sliding window with a length large enough to contain the relevant events (between 100 and 150 ms). The signal contained inside the window was smoothed using a bandpass filter that eliminated all frequencies higher than 70 Hz. This smoothing also reduced the possibility of detecting spurious events by maximizing the noise-signal ratio. The window was selected only if a peak was present at its center and some shape constraints held. These constraints were related to the distribution of the integral of the signal inside the window.

After the CDP candidates were identified, and before generating the basic dictionary of events, we proceeded as follows:

The time-stamp of a detected CDP corresponds to the time of its maximum value in the considered event window. Experts’ knowledge was used to define a suitable time window (*Tw)* around the identified event maximum. In our case, this window had a duration of 100 ms. The signal inside this window was extracted and preprocessed to prepare it for clustering. To this end, we first resampled the signal from 10 to 1.6 kHz for data reduction. In addition, we removed the potential baseline offset by subtracting the average of a subset of the initial points of the signal. This simplified the comparison of different CDPs. Given that the signal must also be sufficiently smooth, the CDPs were processed using PCA as a feature extraction method to compute the most relevant dimensions that describe the whole set of identified CDPs. Finally, only the dimensions that accounted for 98% of the variance were used to reconstruct each CDP. This eliminated all the high-frequency variations in the signal.

To build the dictionary for each signal we used the *k*-means algorithm. The main reason for choosing this algorithm was that resulting cluster prototypes were interpretable and meaningful for the experts. A combination of methods was used for the estimation of the number of clusters required to build the dictionaries to assure the consistency of the result (see Martin et al., 2015). Figures 1G,H shows shape dictionaries obtained from the peaks of signals extracted from the left *L*5 and *L*6 rostral segments. The symbols from these dictionaries were used in the next step to discretize the signals recorded from each segment and time step in the experiment. Each event was associated with the closest cluster in their corresponding dictionary and labeled accordingly. Because there were periods in the signal where no event was detected, a *pause symbol* ($ symbol) was introduced representing this situation. This pause symbol corresponds to parts of the signal not selected and was discarded from the analysis. They do not correspond to a lack of activity but to random fluctuations or events without enough quality to be considered. The duration of this pause symbol was experimentally estimated from the distribution of the time distance among consecutive peaks. The mean of this distribution was used as its duration. If the distance between consecutive events was a multiple of this duration, multiple pause symbols were introduced among them.

### Markovian Behavior of CDPs

In Martin et al. (2015), we showed that the firing of one ensemble of neurons (and so, the appearance of one CDP) depended on the last activated ensemble of neurons (the last appeared CDP). Mathematically, the firing dependence of groups of neurons can be modeled as a Markovian process. With these results, we assumed that the best representation of the dynamical behavior of the recorded sequences are the transition probabilities matrices that describe the behavior as Markov processes of order one. Therefore, we modeled the behavior of lumbar segment *l* in step *s* of the experiment with a matrix *ml,s* consisting in the complete set of transition probabilities between each pair of CDPs:

(5) $$m_{l,s}=\left\{P\left(c_{l,s}^{t+1}=c_{i}|c_{l,s}^{t+1}=c_{j}\right)|\forall c_{i},c_{j}\in CDPs\right\}$$

where probabilities of transitions *P* (*c*_*i*_|*c*_*j*_) were estimated from sequence **C**_*l,s*_ of CDPs recorded in lumbar segment *l* and step *s*. An example of such matrices for two different steps of the experiment in the same lumbar segment is shown in Figures 1G,H.

Finally, we modeled the complete experiment by setting *M*:

(6) $$M=\left\{m_{l,s}|\forall l\in ℒ,s\in S\right\}$$

where ℒ is the set of studied lumbar segments, and *S* is the set of recorded temporal steps. Remember that a temporal step consists of data recorded during the experiment that spans for several minutes, usually 10. Table 1A contains a description of the notation and a description for each entry.

**TABLE 1A:** Description of major symbols used in the text.

**Notation**	**Description**
*L*	Set of lumbar segments
*S*	Set of time steps
*C*_*l,s*_	Sequence of CDPs from lumbar segment l and time step *s*
C${}_{l,s}^{100}$	Subsequence of the last 100 CDPs from sequence C*_*l,s*_*
C${}_{l,s}^{n-100}$	Subsequence CDPs from sequence C*_*l,s*_* without the last 100 CDPs
C${}_{l,s}^{t}$	CDP from a sequence C_*l,s*_ at time instant *t*
*m*_*l*,*s*_	Probability model for lumbar segment l and time step s estimated from a sequence C_*l,s*_ of CDPs
${\hat{m}}_{l,s}$	Probability model for lumbar segment l and time step s estimated from sequence C${}_{l,s}^{n-100}$
*P*(C_*l,s*_|*m_*l,s*_)*	Likelihood of sequence *C*_*l,s*_ for lumbar segment l and time step s given model *M*_*l,s*_
*P*(C*_*l*_*_,s_^*t+1*^|C*_*l*_*_,s_^*t*^;m_*l,s*_)	Probability of CDP *c*${}_{l,s}^{t+1}$ given CDP *c*^*t*^*_*l*_*_,_*_*s*_* and model *m*_*l,s*_
*S(s_*i*_,s_*j*_)*	Similarity index between time step *s*_*i*_ and *s*_*j*_

### Likelihood Computation and Similarity Index Definition

Here, we aimed to obtain a high-level interpretation of the behavior of the potentials recorded in the spinal cord dorsum. We computed for a given lumbar segment *l* the similarity index described above between all pairs of step *s* ∈ *S*. So, for any given step *s* recorded in lumbar segment *l*, we had an index on *how similar* were the data recorded to all other steps, from the Markovian point of view. We decided to visualize that information by depicting a neighborhood graph for the given lumbar segment *l*. In the graph, each node represents a time step of the experiment and edges connect steps of the experiment that are considered *similar*. Following standard procedures to build neighborhood graphs to represent only significant similarities, we allowed connections between nodes only when the following two constraints were fulfilled:

• *k-closest neighbors constraint*: Nodes can only be connected to the *k* most similar nodes. Parameter *k* was set in our experiments in the range [2.10]. Two was the minimum to ensure connections to other nodes. Larger values imply a much more connected network. This constraint avoided graphs overpopulated with too many connections. To set this parameter, we used the method described for the experiment in the previous section. We started from 2 and increased the value of *k* until we found that among the *k* steps with the highest likelihood we recovered the original step in 80% of the cases. According to results shown in Table 1B, it would be *k* = 4.

**TABLE 1B:** Percentage of success using consensus procedure described in section “Consensus Graph Generation” compared with average and random classifier.

	***k***					
Lumbar segment	1	2	3	4	5	6
Random classifier	6	11	17	23	28	32
Average	35	55	71	81	87	90
Consensus	55	82	88	91	91	97

• *Minimum similarity constraint*: Only nodes with similarity higher than a threshold *mst* were connected. Usually, this parameter was set from 0.9 to 0.99, allowing only connections between highly similar nodes. This parameter was used to avoid connections between nodes where a minimum similarity was not achieved. If set to a too high value, all steps were disconnected. To fix this parameter, we started from value 0.99 (from which all nodes are disconnected) and repeatedly reduced the value in 0.01 until each node was connected to at least another one or when we reached the limit of 0.9.

An inspection of the resulting graphs (displayed in Figures 3A,B) shows groups of nodes highly connected among themselves and with few connections with other nodes. However, the structure of the graphs was not evident without a careful examination. In order help to the visualization of the graph’s structure, we applied the methods described in the literature for graph partitioning or clustering (Schaeffer, 2007; Brandes et al., 2008; Fortunato, 2010). Clustering allows a more straightforward interpretation of the graph by separating steps of the experiment with different Markovian behavior. In the graphs depicted in this paper (see Figures 3, 5, 8, 10) we represented clusters of steps of the experiment with the same color. We have experimented with different algorithms obtaining very similar results. However, we decided to use the Walking trap method (Pons and Latapy, 2005), that is based on random walks, because it worked well for relatively small graphs as in this case, and because it allowed building a dendrogram of the obtained clusters for a finely grained study of the results.

### Consensus Graph Generation

For a given experiment, we could examine the graphs obtained for each segment of the spinal cord *l.* However, to obtain a *general vision* of the dynamical behavior of the spinal neuronal network during the experiment, we generated a unique *consensus graph* considering information from all segments. In order to build this consensus graph, we applied ensemble methods, widely used in machine learning (Rokach, 2010). Ensemble methods consider several different predictions of different machine learning models to consensuate one, like in real life one would consider diagnoses of different experts to obtain a reliable outcome. A straightforward way to combine predictions of different models (and very successful in ML) is the simple majority voting (Zhou, 2012). Mathematically, it has been proved that, under general conditions, accuracy in predictions using this technique outperforms prediction and reliability of even the best expert model in the ensemble. So, benefits of following this procedure are not only that we obtain a single representation of the whole behavior of the system but also that we increase the reliability and accuracy of the graph represented.

In our case, we consider each *m*_*l,s*_ a ML model built from data for a particular lumbar segment *l* and step of experiment *s*. Each model predicts (using recorded data) which are the “more similar” steps from the point of view data recorded in that segment (by considering the likelihood of the model *m*_*l,s*_ to generate the data of that step *s*_*t*_). The expression of the majority vote for these models is what we call “consensus graph.” To build this consensus graph we used the following procedure:

As we stated above, we implemented *majority voting* procedure (see Zhou, 2012). We built the consensus graphs by using the *majority voting* scheme as follows:

1. Given a lumbar segment *l*, for each step *s* of that lumbar segment, we made a list with the *k* most similar steps above threshold mst as described in section “Likelihood Computation and Similarity Index Definition.” This list was considered as votes for similar steps of *s* in the lumbar segment *l*.
2. Given a step *s*_*i*_, we collected the votes produced in all lumbar segments *l.* The result was a list of pairs (*s_*j*_, n_*i,j*_)* where *n*_*i,j*_ is the number of votes, that is, the number of lumbar segments for which *s*_*j*_ is among the *k* most similar steps to *s*_*i*_.
3. For each step *s*_*i*_, we kept the *k*2 more voted steps (*k*2 is a new parameter to implement the consensus procedure). In case of ties in votes, we also kept all tied steps. Parameter *k*2 avoids graphs overcrowded with edges and was set to values in the range [2.10]. It plays a role similar to parameter *k* in the building of particular graphs described in the previous section, so it is usually set to *k* or *k-1* value used for specific graphs in point one of this procedure, depending on the number of edges of the final graph.
4. We built a *consensus graph*, with a node for each step of the experiment and edges between steps *s*_*i*_ and *s*_*j*_ if *s*_*j*_ is among the *k*2 most voted steps of *s*_*i*_. As in the case of particular similarity graphs, we did not consider the direction in edges.
5. Finally, the graph was segmented into clusters of nodes using the WalkTrap method as described in the previous section. Nodes belonging to the same cluster were represented with the same color in the figures of the paper.

The consensus graph not only describes the overall behavior of the system, but it also describes it with a degree of reliability higher than each of the individual graphs. One property of the majority vote procedure is that aggregate prediction is more accurate than the average forecast of the events occurring in all the selected segments.
